# Skin Diseases and Syphilis

**Published:** 1894-03

**Authors:** 


### SKIN DISEASES AND SYPHILIS.

EDITED BY DR. M. B. HUTCHINS.

### HYPODERMIC INJECTIONS OF MERCURIAL SALTS IN SYPHILIS.

Moses Endlitz in his thesis comes to the following conclusions (*Jour. des Mal. Cut. et Syph.*):

1. Subcutaneous injections of mercurial salts constitute a good method of treating syphilis.

2. This method has its advantages and disadvantages, from which arise its advantages and disadvantages. It should be employed according to indications, and be neither systematically used nor systematically discarded.

3. It should not be employed in patients affected with kidney troubles or cachexia, whatever be the cause of that cachexia.

4. In other cases it may be employed when patients consent to submit to them.

5. The advantages of injections are: The accuracy of dosage and the rapidity of therapeutic effects.

6. The disadvantages are pain and local reactions, which may be reduced to a satisfactory minimum by a judicious choice of the preparation and a good technique. The cases of death by mercurial intoxication observed as following injections have always been due to medical carelessness.

7. The insoluble preparations are preferable to insure chronic mercurialization. Among these, calomel and the yellow oxide are indicated in grave cases. In other cases, the grey oil or thymolo-acetate are of advantage.

8. Insoluble preparations are to be avoided when the patient cannot be kept under observation, and when he may have his mouth in bad condition.

9. The soluble preparations to be employed are: Corrosive sublimate, peptonate of mercury, biniodide of mercury. The sozoiodolate of mercury is indicated in patients who are not oversensitive.

1. The sozoiodolate of mercury is a very active preparation, but many patients refuse it on account of the painful symptoms it produces.

I am astonished that the bicyanide of mercury is not mentioned. It is very active and efficient in hypodermic injections, which are extremely painful. I have found, however, that the addition of cocaine muriate does not render it less efficient, whilst it makes the injections painless.—*O-D. in St. Louis Med. and Surg. Journal.*

---

TREATMENT OF EPITHELIOMA OF THE FACE. (DR. DARRIER.)

Reviewing the various methods, he describes the following plan which he has found successful.

After removing scales and crusts from the surface, by means of antiseptic poultices, or if the coating is too thick, by touching it with the galvano-cautery, he then anesthetizes the surface, with a 10 per cent. solution of cocaine. Then all the diseased surface is wet by a brush soaked in a concentrated solution of methyl blue one gramme, alcohol and glycerine each 5 grammes. All portions tinted blue are then very lightly touched with a steel probe, wet with a solution of chromic acid 1-5. This produces a purple color. The blue is reapplied, after which the surrounding parts are carefully washed to remove excess of color. The consecutive dressing consists of starch poultices or sublimate compresses, to prevent the formation of crust. The applications are repeated four or five times at intervals of two or three days. Then the blue is used alone until the skin no longer absorbs the color. The treatment lasts from three weeks to two months, for superficial epitheliomas, according to their extent (requiring about one month for each square centimeter). In the deeper forms, interstitial injections of the blue would be indicated, and carefully touching the deeper parts with the chromic acid.

The immediate results are rapid and brilliant. Are they lasting? It is quite probable that relapses will occur, but the treatment is so quickly efficacious and simple, that relapses if they occur may be taken in hand early if the patient is warned of the liability. The author recounts six cases which have been to all appearance permanently cured.—*Bulletin de Therap.*

